# Microglia, Astrocytes, and Oligodendrocytes in Parkinson’s Disease: Neuroinflammatory Crosstalk and Emerging Therapeutic Strategies

**DOI:** 10.3390/biom16010156

**Published:** 2026-01-15

**Authors:** Dominika Kędzia, Grzegorz Galita, Ireneusz Majsterek, Wioletta Rozpędek-Kamińska

**Affiliations:** Department of Clinical Chemistry and Biochemistry, Medical University of Lodz, 92-215 Lodz, Poland; dominika.kedzia@stud.umed.lodz.pl (D.K.); grzegorz.galita@umed.lodz.pl (G.G.); ireneusz.majsterek@umed.lodz.pl (I.M.)

**Keywords:** microglia, astrocytes, oligodendrocytes, neuroinflammation, crosstalk, Parkinson’s disease, therapy

## Abstract

Parkinson’s disease (PD) is a progressive neurodegenerative disorder marked by the degeneration of dopaminergic neurons in the substantia nigra, resulting in cardinal motor symptoms such as tremor, rigidity, and bradykinesia. Neuroinflammation is increasingly recognized as a central driver of PD onset and progression in which oligodendrocytes, astrocytes, and microglia engage in complex bidirectional crosstalk that shapes the inflammatory milieu of the central nervous system. Pathological activation of glial cells triggers the release of pro-inflammatory cytokines, chemokines, and reactive oxygen species, thereby exacerbating neuronal injury and contributing to sustained disease progression. Modulating maladaptive glial activation states and their intercellular communication represents a promising therapeutic avenue aimed at mitigating neuroinflammation and slowing PD pathology. This review synthesizes current knowledge on neuroinflammation in PD, focusing on the distinct roles of microglia, astrocytes, and oligodendrocytes, their interaction networks, and emerging therapeutic strategies.

## 1. Introduction

Parkinson’s disease (PD) is a progressive neurodegenerative disorder defined by the selective degeneration of dopaminergic neurons within the substantia nigra pars compacta (SNpc), a midbrain structure essential for the regulation of motor function [1,2]. Its etiopathogenesis reflects a multifaceted convergence of genetic susceptibility, environmental exposures, and cellular dysfunctions that collectively perturb neuronal integrity [3]. A central pathological feature of PD is the misfolding and aggregation of the presynaptic protein α-synuclein (α-syn), which assembles into insoluble fibrils that accumulate as Lewy bodies and Lewy neurites, progressively impairing synaptic signaling and ultimately promoting neuronal death [4,5,6,7,8]. The resulting degeneration of dopaminergic projections leads to dopamine deficiency and manifests clinically as tremor, rigidity, bradykinesia, and postural instability, while a broad spectrum of non-motor symptoms—including olfactory impairment, sleep disturbances, autonomic dysfunction, psychiatric manifestations, and cognitive decline—frequently accompany disease progression [9].

α-Synuclein aggregation is mechanistically linked to mitochondrial dysfunction, oxidative stress, lysosomal failure, and global disturbances in cellular homeostasis. These pathological cascades converge on robust neuroinflammatory responses, now recognized as major determinants of both the onset and progression of PD [5,10,11,12]. Neuroinflammation is driven primarily by the activation of microglia and astrocytes, which release pro-inflammatory cytokines, chemokines, reactive oxygen species, and additional mediators that exacerbate ongoing neuronal injury [4,13]. Cytokines can provide early insight into the progression of PD and treatment responses as potential biomarkers. Beyond classical glial cytokines, several other inflammation-linked biomarkers have been identified in PD, including sensitivity C-reactive protein (hsCRP), and TNF-α/soluble TNF receptors (sTNFRs) or chemokines [14].

Microglia, the resident macrophages of the central nervous system (CNS), rapidly shift toward classically described pro-inflammatory (M1) or anti-inflammatory (M2) phenotypes, thereby orchestrating local immune responses. Astrocytes similarly undergo reactive transformation into neurotoxic (A1) or neuroprotective (A2) states and maintain tight reciprocal communication with microglia [15]. Beyond these two glial populations, oligodendrocytes have recently gained recognition as active participants in PD-related neuroinflammation; rather than serving solely supportive or passive roles, they can contribute directly to inflammatory signaling via glia–neuron communication pathways [16].

Increasing evidence highlights the existence of a highly integrated crosstalk network among oligodendrocytes, astrocytes, and microglia, collectively shaping the inflammatory landscape of the PD brain [17,18]. Deciphering the molecular mechanisms that regulate this intercellular communication is essential for identifying actionable therapeutic targets. Modulating maladaptive glial activation states and disrupting pathological glial crosstalk represent promising strategies to mitigate neuroinflammation. Ferroptosis plays a significant role in neuroinflammation. In Parkinson’s disease, ferroptosis in glial cells contributes to neuroinflammation and the loss of dopaminergic neurons by promoting lipid peroxidation, iron dysregulation, and the release of cytokines [19]. The ferroptosis of microglia and astrocytes amplifies inflammatory signaling, while dysfunction in oligodendrocytes may extend ferroptotic stress to nearby glia and neurons [20]. Additionally, reciprocal signaling between oligodendrocytes and astrocytes under oxidative stress further propagates ferroptosis by impairing iron metabolism and antioxidant pathways [21]. In this context, glial ferroptosis is both a result of inflammation and a contributor to it. Oxidative stressors activate glial cells and lead to cell death, while dying glial cells create an inflammatory environment that accelerates the degeneration of nearby neurons [22]. This glia-centered perspective on ferroptosis suggests potential new therapeutic strategies [23].

## 2. Role of Microglia in PD Pathogenesis

Microglia, the resident immune cells of the central nervous system, support neuronal function by pruning synapses, remodeling neural circuits, and clearing damaged cells and protein aggregates through phagocytosis. They also maintain tissue homeostasis by regulating inflammatory signaling via the release of cytokines and chemokines [18,24,25]. In the context of PD, microglia exert dual roles, contributing both to neuroprotection and to disease progression [26,27]. They readily recognize and respond to α-synuclein aggregates; however, such activation may either promote α-synuclein clearance or drive maladaptive microglial states characterized by a glycolytic metabolic shift, mitochondrial dysfunction, and upregulation of inducible nitric oxide synthase (iNOS) [28,29]. α-Synuclein engagement further impairs microglial phagocytosis, increases the production of reactive oxygen species (ROS), and induces oxidative stress. Accumulation of α-synuclein within microglia ultimately exhausts their protective capacity and propagates toxic inflammation, thereby promoting neurodegeneration [30].

### Role of Microglia in Neuroinflammation

Microglia are traditionally categorized into pro-inflammatory “M1” and anti-inflammatory “M2” states; however, this dichotomy oversimplifies a broad continuum of context-dependent phenotypes observed in PD [31]. The traditional M1/M2 paradigm is increasingly viewed as an oversimplified and outdated model that does not adequately capture the dynamic and context-dependent heterogeneity of microglial activation states [15]. Transcriptomic studies reveal multiple microglial subpopulations defined by distinct marker-gene signatures that reflect diverse activation states [15,32]. Reactive microglia undergo profound morphological, molecular, and functional remodeling in response to pathological cues such as α-synuclein deposition [15].

In PD, microglial exposure to α-synuclein induces protein aggregation within these cells and elicits robust activation characterized by hypertrophic morphology, enlarged soma, and increased expression of activation markers [30]. Microglia detect misfolded α-synuclein—which acts as a damage-associated molecular pattern (DAMP)—through pattern recognition receptors (PRRs), including Toll-like receptors (TLRs) and Fcγ receptors (FcγRs) [29,33]. Toll-like receptor 4 (TLR4) activation by extracellular α-synuclein triggers nuclear factor-kappa B (NF-κB) signaling and upregulates the autophagy receptor p62/sequestosome-1 (SQSTM1), initiating synucleinphagy—a selective autophagic pathway that degrades ubiquitinated α-synuclein [34]. Despite these protective mechanisms, microglia heavily burdened with α-synuclein show impaired phagocytosis, promote oxidative damage, and recruit peripheral immune cells producing interferon-γ (IFN-γ), creating a self-amplifying inflammatory loop [30].

TLR4 is also implicated in mediating neuroinflammation across multiple CNS cell types, and its co-localization with pathological α-synuclein may reflect glial activation following neuronal injury [35]. In parallel, α-synuclein oligomers and fibrils enhance the interaction between Toll-like receptor 2 (TLR2) and its adaptor myeloid differentiation factor 88 (MyD88), promoting NF-κB activation and cytokine production and driving a pro-inflammatory phenotype [36].

Activation of NF-κB and other transcription factors downstream of PRRs primes the NOD-like receptor protein 3 (NLRP3) inflammasome [37,38,39]. Priming can be triggered by α-synuclein or lipopolysaccharide (LPS), leading to the release of tumor necrosis factor-α (TNF-α), interleukin-1β (IL-1β), and interleukin-6 (IL-6) via converging molecular pathways [40,41]. Activation of NLRP3 in human primary microglia promotes caspase-1 recruitment and subsequent secretion of IL-1β and interleukin-18 (IL-18) [42,43]. Microglial activation also leads to the recruitment of peripheral immune cells through chemokines such as C-C motif chemokine ligand 2 (CCL2) [44]. Aggregated α-synuclein additionally induces IL-1β secretion via cluster of differentiation 36 (CD36)-Fyn signaling, where Fyn facilitates priming through protein kinase C delta (PKCδ)–NF-κB and triggers NLRP3 activation via mitochondrial reactive oxygen species (ROS), culminating in caspase-1–dependent IL-1β maturation [45]. Nevertheless, microglia also secrete anti-inflammatory cytokines such as interleukin-4 (IL-4) and interleukin-10 (IL-10), which alleviate neuroinflammation and support dopaminergic neuron survival [26].

Reactive oxygen species critically regulate NLRP3 activation, with contributions from both mitochondrial superoxide and nicotinamide adenine dinucleotide phosphate (NADPH) oxidase–derived ROS. Inhibition of NADPH oxidases, particularly the NADPH oxidase 2 (NOX2) subunit, reduces ROS production and mitigates NLRP3-dependent neuroinflammation [46]. Oxidative stress additionally activates NF-κB signaling, amplifying pro-inflammatory cytokine expression [47]. ROS generated via iron-dependent lipid peroxidation contribute to ferroptosis, particularly during glutathione (GSH) depletion and glutathione peroxidase 4 (GPX4) dysfunction. Microglia facilitate ferroptosis-related neurodegeneration; although early iron sequestration may confer protection, microglial ferroptotic death generates neurotoxic conditions. Interleukin-8 (IL-8) and IL-1β have been implicated as contributors to post-ferroptotic inflammation [20]. Ferroptosis also influences non-coding RNAs. In the nuclear enriched abundant transcript 1 (NEAT1)/microR-26b-5p (miR-26b-5p)/S100 calcium-binding protein A2 (S100A2) axis, NEAT1 knockdown decreases miR-26b-5p and S100A2, reduces ROS, and regulates the progression of ferroptosis [48].

Non-coding RNAs (ncRNAs) exert key regulatory functions in microglial inflammatory responses in PD [49,50,51]. Several long non-coding RNAs (lncRNAs) are upregulated during microglial activation and act as competing endogenous RNAs (ceRNAs), sequestering anti-inflammatory microRNAs (miRNAs) and thereby sustaining pro-inflammatory signaling [52]. For example, long non-coding RNA growth arrest specific 5 (lncRNA GAS5) functions as a molecular sponge for microRNA-233-3p (miR-223-3p), a negative regulator of NLRP3, promoting aberrant inflammasome activation [53]. Conversely, certain ncRNAs exhibit neuroprotective effects; the microRNA-124 (miR-124) attenuates microglia-mediated neuroinflammation by inhibiting the mitogen-activated protein kinase kinase kinase 3 (MEKK3)/NF-κB axis and suppressing pro-inflammatory gene transcription [52].

## 3. Role of Astrocytes in PD Pathogenesis

Astrocytes are multifunctional glial cells that provide essential support to neuronal networks. They regulate synaptic transmission by clearing glutamate and γ-aminobutyric acid (GABA), secrete neurotrophic factors that promote neuronal survival, maintain ion homeostasis, and contribute to the integrity of the blood–brain barrier (BBB). In addition, astrocytes support neuronal resilience by secreting antioxidant and neurotrophic molecules, removing extracellular α-synuclein, regulating glutamate and fatty acid metabolism, and transferring functional mitochondria to compromised neurons [54,55].

Astrocytic dysfunction is increasingly recognized as a key contributor to PD pathogenesis. Impaired astrocytes show reduced neurotransmitter clearance, dysregulated intracellular calcium signaling, and diminished neurotrophic and metabolic support [56,57]. These deficits promote excitotoxicity and oxidative stress, ultimately facilitating dopaminergic neuron degeneration [54,58]. Astrocytes also play a dual role in modulating α-synuclein pathology: while they can internalize and degrade α-synuclein, they may also propagate its aggregates and activate pro-inflammatory signaling pathways, thereby contributing to chronic neuroinflammation [59,60,61].

### Role of Astrocytes in Neuroinflammation

Reactive astrogliosis encompasses a spectrum of morphological and functional changes that astrocytes undergo in response to injury or neurodegeneration [62]. Historically, reactive astrocytes have been grouped into pro-inflammatory “A1” and neuroprotective “A2” states [61]. Recent transcriptomic data, however, demonstrate that astrocyte reactivity is highly heterogeneous, and PD astrocytes may simultaneously express both A1 and A2 markers [61].

Although Lewy bodies primarily accumulate in neurons, α-synuclein aggregates have also been detected within astrocytes in postmortem PD brain tissue [63]. Bidirectional transfer of α-synuclein between neurons and astrocytes contributes to neurodegeneration, with astrocytes capable of shuttling α-synuclein via endocytosis or extracellular vesicles [64,65]. Astrocyte–astrocyte transfer may further occur via tunneling nanotubes, enabling the spread of both α-synuclein and major histocompatibility complex class II (MHC-II) [66,67]. Oligomeric α-synuclein induces astrocytic secretion of vascular endothelial growth factor A (VEGFA) and nitric oxide (NO), thereby promoting BBB disruption in PD [68]. Complement component C4, elevated in PD, further amplifies neuroinflammation by activating astrocytes and enhancing cytokine release in response to α-synuclein preformed fibrils (PFFs), leading to neuronal toxicity [69].

Astrocytes also contribute to α-synuclein pathology through Toll-like receptor signaling. TLR2 activation in both neurons and astrocytes suppresses autophagy and reduces α-synuclein clearance, promoting A1-like neurotoxic reactivity marked by the upregulation of SerpinG1, complement component C3, proteasome subunit beta type-8 (PSMB8), and guanylate-binding protein 2 (GBP2) [70]. Additionally, the impact of α-syn on TLR4 astrocytes induces the nuclear translocation of p65, thereby enhancing NF-κB-dependent transcription [71].

Activated astrocytes modulate inflammatory pathways, including NF-κB and janus kinase (JAK)/signal transducer and activator of transcription (STAT), which induce the expression of pro-inflammatory cytokines (IL-1, IL-6, TNF-α) and chemokines—C-X-C motif chemokine ligand 1 (CXCL1) and C-X3-C motif chemokine ligand 1 (CX3CL1) [72]. The JAK2/signal transducer and activator of transcription 3 (STAT3) pathway is a central mediator of astrocyte reactivity. Cytokines such as IL-6 activate JAK2/STAT3 in astrocytes, driving astrogliosis [73,74]. Single-nucleus RNA sequencing of PD substantia nigra reveals upregulation of STAT3-dependent gene networks, while spatial transcriptomics identifies cluster of differentiation 44 (CD44^+^) reactive astrocytes enriched in inflammatory pathways [75]. CD44 knockdown suppresses JAK/STAT activation, demonstrating its role in astrocyte-mediated inflammation [75].

NF-κB regulates numerous pro-inflammatory genes [61]. Toll-like receptors such as TLR4 detect DAMPs, LPS, or α-synuclein and initiate NF-κB signaling in astrocytes [76,77]. As in microglia, α-synuclein can activate the NLRP3 inflammasome in astrocytes, promoting caspase-1–mediated release of IL-1β and IL-18. Autophagy protein 5 (Atg5) modulates this response, as pharmacological suppression of autophagy inhibits IL-1β secretion [78]. Astrocytic cannabinoid receptor 2 (CB2R) activation attenuates NLRP3 inflammasome signaling by suppressing fork head box g1 (Foxg1)-dependent repression of microtubule-associated protein 1 light chain 3 beta (MAP1LC3B), thereby enhancing autophagy-mediated NLRP3 degradation. In 1-methyl-4-phenyl-1,2,3,6-tetrahydropyridine (MPTP)-based PD models, this mechanism reduces neuroinflammation and protects dopaminergic neurons [79]. Also, mitochondrial stress influences on NRLP3 and active NLRP3 exacerbate mitochondrial injury [80].

Astrocytic mitochondrial dysfunction is an important driver of chronic oxidative stress and neuroinflammation in PD [81]. NADPH oxidase 4 (NOX4), a generator of hydrogen peroxide (H_2_O_2_), is elevated in PD and promotes inflammatory cytokine production, myeloperoxidase (MPO) activity, and osteopontin (OPN) expression [82]. These mediators impair mitochondrial electron transport chain complexes and increase 4-hydroxynonenal (4-HNE) levels, leading to astrocytic ferroptosis [82]. Ferroptosis contributes to iron deposition, excessive ROS production, iron-dependent lipid peroxidation, and defective lipid peroxide clearance [83]. Moreover, ncRNAs can affect the process of ferroptosis [48].

Non-coding RNAs are additional regulators of astrocyte-mediated neuroinflammation in PD [84]. Dysregulated miRNAs influence inflammatory cascades and cytokine production [85]. Astrocytes in PD adopt reactive states that promote inflammation; however, miR-29b2/c deficiency enhances AMP-activated protein kinase (AMPK) activity while suppressing NF-κB/p65 signaling in glial cells [86]. Deletion of the miR-29a/b1 locus reprograms astrocytes toward a less pro-inflammatory, AMPK-high phenotype characterized by reduced A1 gene expression, decreased TNF-α/IFN-γ/monocyte chemoattractant protein-1 (MCP-1) secretion, and increased production of neurotrophic factors such as brain-derived neurotrophic factor (BDNF) and transforming growth factor-β 1 (TGF-β1) [87]. In MPTP PD models, this shift reduces glial fibrillary acidic protein (GFAP)-positive astrogliosis, preserves nigrostriatal dopaminergic neurons, and improves motor performance, underscoring miR-29a/b1 as an important regulator of astrocyte-mediated neuroprotection [87].

## 4. Role of Oligodendrocytes in PD Pathogenesis

Oligodendrocytes are central nervous system glial cells responsible for generating myelin, which insulates axons and enables rapid saltatory conduction [88]. Although dopaminergic neurons of the substantia nigra possess only lightly myelinated axons, they rely on oligodendrocytes for critical structural and metabolic support [89]. Notably, the distribution of oligodendrocyte precursor cells (OPCs) within the SNpc does not correlate with myelin basic protein (MBP) expression or myelin distribution [90]. Instead, OPCs are evenly dispersed throughout the SNpc, interacting with neuronal somata as well as axons [90].

Growing evidence indicates that oligodendrocytes may play a previously underappreciated role in PD pathogenesis. In the striatum—particularly within the putamen—oligodendrocytes display pronounced molecular and cellular abnormalities, including transcriptional dysregulation linked to cellular stress responses, protein misfolding, and impaired myelination [89]. Their intrinsic vulnerability stems from high metabolic activity and elevated iron content, coupled with relatively weak antioxidant defenses. Thus, oxidative stress, a hallmark of PD pathology, promotes oligodendrocyte injury and death, exacerbating neuronal dysfunction [23,88].

### Role of Oligodendrocytes in Neuroinflammation

Chronic neuroinflammation is a defining feature of PD. Historically, oligodendrocytes were not considered key contributors to inflammatory responses, yet emerging evidence suggests that they actively modulate the neuroinflammatory milieu [88]. Oligodendrocytes express cytokine- and damage-associated receptors capable of initiating intracellular signaling cascades and influencing cytokine secretion [91]. Nevertheless, their immunological role in PD remains insufficiently characterized.

Transcriptomic studies show that oligodendrocytes in PD upregulate genes associated with stress responses, inflammation, and the unfolded protein response, while downregulating genes essential for myelination [89,92,93,94]. Single-nucleus RNA sequencing further demonstrates that oligodendrocytes endogenously express SNCA, the gene encoding α-synuclein [88]. Complementary in vitro findings indicate that oligodendrocytes can internalize extracellular α-synuclein, implicating them in its potential propagation [88].

Moreover, oligodendrocytes in PD contain juxtanuclear mercury deposits, which may accelerate α-synuclein aggregation, membrane injury, mitochondrial and lysosomal dysfunction, and oxidative stress [95]. Accumulation of α-synuclein within oligodendrocytes exerts cytotoxic effects, disrupts myelin maintenance, and promotes oligodendroglial cell death [96,97]. In patient-derived induced pluripotent stem cell (iPSC) oligodendrocytes from PD and multiple system atrophy (MSA), both endogenous α-synuclein overexpression and uptake of extracellular α-synuclein trigger extensive transcriptional reprogramming. This response features reduced expression of myelin and maturation-related genes alongside increased expression of major histocompatibility complex class I (MHC-I), MHC-II, and interferon pathways components [98].

Oligodendrocytes are also sensitive to ferroptosis, a form of iron-dependent, lipid peroxidation–driven cell death exacerbated by PD-associated redox abnormalities. Increased ROS levels, dysregulated iron metabolism, and altered lipid homeostasis promote oligodendrocyte loss via ferroptosis, contributing to neuronal vulnerability [99]. IL-1β further modulates iron handling in oligodendroglial cells: in undifferentiated MO3.13 cells, IL-1β elevates iron regulatory protein 1 (IRP1) and transferrin receptor 1 (TfR1), while reducing ferroportin (FPN1), thereby promoting iron accumulation [100]. In differentiated oligodendrocytes, IL-1β reduces TfR1 and increases FPN1 expression, enhancing iron efflux. These bidirectional effects alter iron homeostasis, potentially impairing oligodendrocyte maturation and contributing to dopaminergic neuron damage in PD [100].

Recent studies reveal a pathogenic signaling axis between dopaminergic neurons and oligodendrocytes. Dopaminergic neurons secrete prosaposin (PSAP), which activates G-protein–coupled receptor 37 (GPR37) on oligodendrocytes, stimulating IL-6 production [16]. Notably, genetic deletion of oligodendrocytic GPR37 or short hairpin RNA (shRNA)-mediated IL-6 knockdown prevents dopaminergic neuron loss, motor impairment, and chronic pain in PD mouse models, implicating oligodendrocyte-derived IL-6 as a key mediator of neurodegeneration [16].

## 5. Crosstalk Between Oligodendrocytes, Microglia and Astrocytes in Neuroinflammation in PD

**Figure 1:**
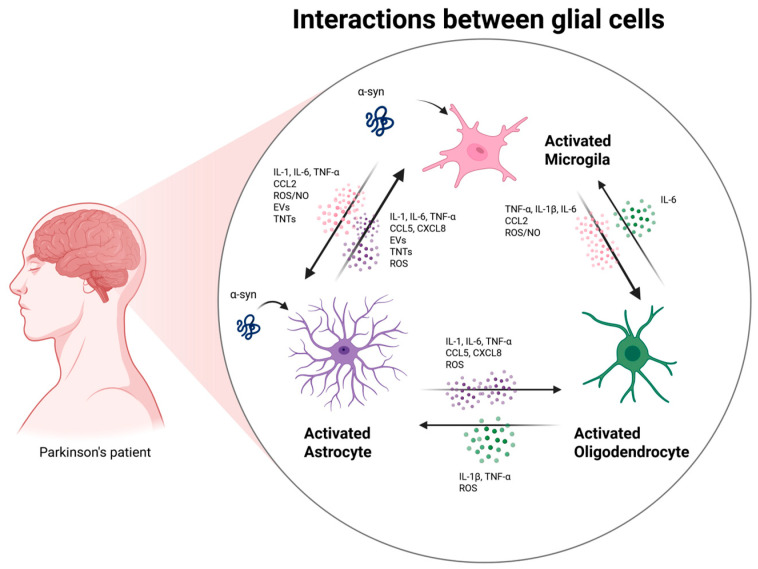
In PD, microglia, astrocytes, and oligodendrocytes engage in a tightly interconnected proinflammatory crosstalk mediated by cytokines, chemokines, ROS/NO, and extracellular vesicles (EVs) [104]. Glial cells can be activated by α-synuclein, which affects the activation of signaling pathways [33]. Activated microglia release IL-1β, TNF-α, chemokines, and oxidative mediators that convert astrocytes into reactive phenotypes and propagate inflammatory signaling [33]. Reactive astrocytes amplify this network by secreting IL-1, IL-6, TNF-α, chemokines, and ROS, while also responding to and redistributing microglial EV cargo rich in inflammatory molecules [72]. Oligodendrocytes, once considered passive, participate in this bidirectional exchange by producing IL-6 and other cytokines in response to microglial and astrocytic cues, thereby reinforcing glial activation and disrupting homeostatic support [16]. This cell’s crosstalk creates a neuroinflammation feedback loop that enhances inflammation in Parkinson’s disease and influences the progression of the condition [104].

The interplay among microglia, astrocytes, and oligodendrocytes forms an integrated regulatory network that shapes the inflammatory milieu in the CNS during Parkinson’s disease (Figure 1). In PD, all three glial cell types undergo reactive transformation and engage in intensive bidirectional communication, establishing a self-perpetuating inflammatory loop that exacerbates neuronal dysfunction and degeneration. This glial crosstalk amplifies the release of pro-inflammatory mediators, oxidative stress, and metabolic dysregulation, but it may also represent a set of regulatory checkpoints with therapeutic potential [96,101,102,103].

### 5.1. Microglia–Astrocyte Crosstalk

#### 5.1.1. Role of LPS in Neuroinflammation

The microglial NLRP3 inflammasome, activated downstream of LPS signaling, plays a pivotal role in inducing neurotoxic A1-type astrocytes through NF-κB–dependent cytokine release and caspase-1 activation [41]. Early LPS-induced neuroinflammation is characterized by concurrent NLRP3 activation, complex I mitochondrial dysfunction, and elevated nitrosative and oxidative stress, collectively shaping the neuroinflammatory landscape of PD [41].

#### 5.1.2. Influence of α-Syn on Microglia–Astrocyte Interactions

Although both cell types respond to extracellular α-syn via TLR activation, microglia exhibit broad TLR expression, while astrocytes display comparatively restricted TLR profiles. As a result, astrocytes largely depend on microglia to detect pathogenic stimuli and initiate intercellular inflammatory signaling, underscoring the centrality of microglia–astrocyte crosstalk in CNS inflammatory responses. Depending on the activation state of microglia, these signals can lead astrocytes toward neurotoxic, pro-inflammatory phenotypes or promote anti-inflammatory feedback mechanisms [15].

Pathological α-synuclein oligomers, rather than monomeric forms, serve as potent triggers of microglia–astrocyte crosstalk [105]. These oligomers activate microglia and astrocytes via NF-κB signaling and induce upregulation of astrocytic T-type Ca^2+^ channels (Ca_v_3.2), thereby altering calcium dynamics and the astrocytic secretome, including Insulin-like growth factor binding protein like 1 (IGFBPL1) [105]. This dual modulation may contribute to both neuroinflammatory and neuroprotective functions of astrocytes in synucleinopathies [105].

Cntnap4 deficiency worsens α-syn pathology by promoting mitochondrial dysfunction, ferroptosis, and increased α-syn release in dopaminergic neurons [106]. The resulting neuronal stress activates a microglia–astrocyte inflammatory loop mediated by the complement C3 (C3)–C3a receptor (C3aR) complement pathway [106]. Astrocyte activation toward an A1 phenotype enhances C3 production, which promotes opsonization of α-syn aggregates and exacerbates inflammation [107].

Extracellular α-syn activates nucleotide-binding oligomerization domain containing 2 (NOD2) in microglia, promoting receptor-interacting serine/threonine-protein kinase 2 (RIPK2) signaling and mitogen-activated protein kinase (MAPK)/NF-κB pathway activation, ultimately triggering reactive astrocyte formation and dopaminergic neuron degeneration [108]. Additionally, NOD-like receptor CARD domain–containing 5 (NLRC5) in microglia and astrocytes promotes pro-inflammatory signaling. Deficiency of NLRC5 suppresses nuclear factor kappa B (NF-κB) and mitogen-activated protein kinase (MAPK) pathways while enhancing protein kinase B (AKT)–glycogen synthase kinase 3 beta (GSK-3β) and adenosine monophosphate–activated protein kinase (AMPK) signaling [109].

**Figure 2:**
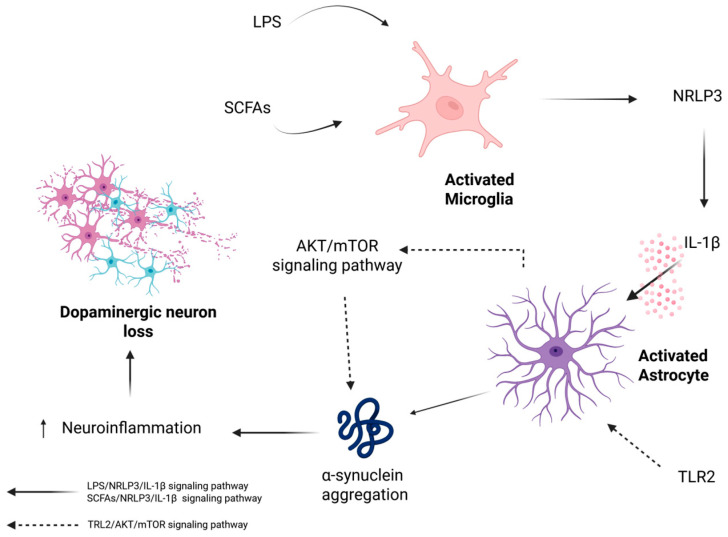
Neuroinflammatory signals can lead to α-synuclein accumulation in glial cells by impairing its clearance through the activation of astrocytic TLR2–AKT–mTOR signaling [44], as well as by increasing its expression via IL-1β-driven upregulation of α-synuclein [67]. Additionally, the activation of the microglial NLRP3 inflammasome by LPS or SCFAs increases the levels of IL-1β, which contributes to neuroinflammation [40,111]. This process can further promote α-synuclein aggregation and result in the loss of dopaminergic neurons [40,111].

On the other hand, α-syn can be accumulated in glial cells under the influence of cytokines. Neuroinflammatory mediators and immune cell infiltration exacerbate the impact of α-synuclein on disease progression, which occurs before the loss of nigrostriatal dopaminergic neurons [110]. TLR2 activation in astrocytes may inhibit the clearance of α-synuclein through the AKT- mammalian target of rapamycin (mTOR) signaling pathway, leading to increased aggregation of α-synuclein [44]. Furthermore, in astrocytes, α-syn expression is increased by astrocyte exposure to interleukin-1β (IL-1β), suggesting that external stimuli affect astrocyte α-syn expression [67]. Microglia can release IL-1β during the activation of NLRP3 by LPS [40], potentially influencing α-syn expression in astrocytes. NLRP3 can also be activated by short-chain fatty acids (SCFAs) through the SCFA/G protein–coupled receptor 43 (GPR43)-NLRP3 pathway [111]. SCFAs influence α-synuclein aggregation, dopaminergic neuronal loss, and inflammatory responses [111] (Figure 2).

#### 5.1.3. Tunneling Nanotubes, Extracellular Vesicles, and Secretion of Mitochondrial-Derived Vesicles in α-Synuclein Microglia–Astrocyte Crosstalk Transfer and Neuroinflammatory Amplification

Tunneling nanotubes (TNTs) represent direct cytoplasmic conduits enabling intercellular transfer of α-syn fibrils, organelles, and signaling molecules between glial cells. Astrocytes exposed to α-syn generate transient TNTs that facilitate the transfer and degradation of α-syn fibrils [17,112]. However, excessive α-syn burden can overwhelm microglial clearance mechanisms, revealing the limits of their protective capacity and highlighting their potential pathogenic contribution [44]. TNT-mediated intercellular exchange additionally reduces intracellular ROS accumulation, indicating that glial communication can exert both protective and detrimental effects [113,114].

Extracellular vesicles (EVs), including exosomes, constitute another major mode of glial communication. EVs carry lipids, proteins, miRNAs, and α-syn oligomers, propagating inflammatory signals between glial populations [115,116,117]. Exosomes released by α-syn PFF–activated microglia promote the conversion of astrocytes into a neurotoxic phenotype by delivering inflammatory mediators and α-syn oligomers [118]. This process is regulated by the microglial E3 ligase Peli1, which governs microglial activation and EV release. Reactive astrocytes subsequently enhance Peli1 expression in microglia, reinforcing a self-sustaining inflammatory loop [118].

Moreover, mitochondrial dysfunction impairs mitochondrial quality control and reduces the secretion of mitochondrial-derived vesicles (MDVs) [117]. Accumulation of damaged mitochondria leads to mitochondrial DNA (mtDNA) and other DAMP release via exosomes, activating TLRs, the NLRP3 inflammasome, and the cyclic GMP-AMP synthase (cGAS)–stimulator of interferon genes (STING) pathway [117]. These findings link defective PTEN-induced kinase 1 (PINK1)/Parkin-mediated MDV formation to innate immune activation and systemic propagation of PD pathology [117].

#### 5.1.4. Role of Cytokines and Chemokines

Microglia and astrocytes engage in extensive cytokine and chemokine exchange that amplifies neuroinflammation. Astrocytic cytokine release—facilitated by aquaporin-4 (AQP4)—propagates inflammatory signaling and promotes microglial activation [119]. Conversely, microglia induce neurotoxic A1 astrocytes through interleukin-1α (IL-1α), TNF-α, and complement C1q, which drive transcriptional and morphological reactivity [120]. IL-1β and TNF-α co-stimulation enhances astrocytic GFAP, vimentin, C3, C-C motif chemokine ligand 5 (CCL5), C-X-C motif chemokine ligand 8 (CXCL8), and lipocalin 2 (LCN2) expression, promoting a robust pro-inflammatory phenotype [121].

Microglia show higher baseline expression of several chemokine receptors, including C-X-C motif chemokine receptor 1 (CXCR1), C-X-C motif chemokine receptor 3 (CXCR3), and C-C chemokine receptor 3 (CCR3), whereas astrocytes typically express more moderate levels of CXCR1 and CXCR3 and lower levels of additional receptors [122]. Under pro-inflammatory conditions, astrocytes upregulate chemokine receptors, particularly CXCR3, CCR3, and CXCR1, consistent with increased chemokine sensitivity during neuroinflammation [122].

CXCR4 deletion reduces dopaminergic neuron loss, microglial and astrocytic activation, cytokine expression, peripheral immune infiltration, and blood–brain barrier disruption, demonstrating that CXCR4 signaling contributes to PD-associated neuroinflammation [123]. Conversely, interleukin-33 (IL-33) supports microglia–astrocyte communication and suppresses glial activation in vitro, protecting neurons from injury [124].

### 5.2. Astrocyte–Oligodendrocyte Crosstalk

Astrocytes play a critical role in supporting the maturation and survival of newly formed myelin-producing oligodendrocytes. During efficient remyelination, astrocytes downregulate the nuclear factor erythroid 2-related factor 2 (Nrf2) pathway while simultaneously upregulating cholesterol biosynthesis and efflux, processes essential for promoting oligodendrocyte survival [125]. Both astrocytes and oligodendrocytes contribute to the neuroinflammatory environment by producing immunoregulatory cytokines, thereby influencing inflammation-mediated injury and repair [126].

#### 5.2.1. Role of Cytokines in Astrocyte–Oligodendrocyte Crosstalk

α-Synuclein inclusions occur in both astrocytes and oligodendrocytes in Parkinson’s disease, with their burden correlating with disease progression. While α-syn pathology in oligodendrocytes progresses similarly to that observed in neurons, astrocytic pathology appears later and follows a distinct temporal pattern [127]. Accumulating α-synuclein contributes to neuroinflammation and cytokine production.

Reactive astrocytes secrete pro-inflammatory cytokines, including IL-6, TNF-α, and IL-1β, as well as chemokines that can damage both neurons and oligodendrocytes [103]. These cytokines promote demyelination, hypomyelination, oligodendrocyte necrosis, and apoptosis [103]. Conversely, oligodendrocytes release modulatory mediators such as CCL2, which downregulate astrocytic IL-6 expression and thus reduce inflammatory signaling [103,128].

Oligodendrocyte-derived IL-6 also contributes to PD neuroinflammation. PSAP activates GPR37 on oligodendrocytes, inducing IL-6 upregulation and secretion, which in turn exacerbates neuroinflammation [16]. Experimental IL-6 overexpression in the substantia nigra induces microglial and astrocytic activation as well as dopaminergic neuron loss, highlighting the role of oligodendrocyte-derived cytokines in glial-mediated neurotoxicity [16].

#### 5.2.2. Neuregulin-Mediated Signaling Pathway on Neuroinflammation

The Neuregulin-1 (NRG1)/ErbB signaling pathway, long recognized for its developmental functions, is increasingly implicated in glial communication during neurodegeneration [129]. NRG1 activates ErbB family receptor tyrosine kinases—particularly ErbB3 and ErbB4—triggering intracellular cascades involved in survival and glial function [129]. Astrocytes and oligodendrocytes express components of this pathway, including ErbB1, ErbB2, and ErbB3 [129].

Single-cell computational analyses of PD brain tissue reveal active astrocyte–OPC communication through NRG1–ERBB4 signaling [130]. Astrocytes act as both ligand producers and signal receivers, whereas OPCs express complementary ligands, establishing a bidirectional axis of glial interaction. This NRG1-dependent communication likely contributes to neuroinflammation, glial structural support, and neurodegenerative processes in PD [130].

#### 5.2.3. Role of Fibroblast Growth Factor (FGF) Signaling Pathway in Neuroinflammation

Single-nucleus RNA sequencing combined with spatial transcriptomics in PD mouse models demonstrates disrupted interglial communication, particularly involving FGF signaling [21]. Signaling via fibroblast growth factor 1 (FGF1), fibroblast growth factor 9 (FGF9), and receptors fibroblast growth factor receptor 1 (FGFR1), fibroblast growth factor receptor 2 (FGFR2), and fibroblast growth factor receptor 3 (FGFR3) is reduced between oligodendrocytes and astrocytes [21]. Impaired FGF-mediated crosstalk enhances glial ferroptotic activity, elevates mitochondrial oxidative phosphorylation and ROS production in both cell types, and contributes to neuroinflammation [21].

Astrocytes exhibit elevated intracellular Ca^2+^, diminished Mt1 expression, and iron accumulation within the substantia nigra, accompanied by downregulation of the NRF2/solute carrier family 7 member 11 (SLC7A11)/GPX4 antioxidant axis [99]. Immune activation and calcium dysregulation promote ferroptosis-mediated degeneration of both neurons and oligodendrocytes [99]. Given their high iron content and limited antioxidant capacity, oligodendrocytes are particularly susceptible to oxidative injury [21].

### 5.3. Microglia–Oligodendrocyte Crosstalk

Microglia–oligodendrocyte interactions play an important but understudied role in the pathogenesis of Parkinson’s disease. Microglial phenotype significantly regulates neuron-glia antigen 2 (NG2-glia)—oligodendrocyte precursor cells, influencing their proliferation, migration, and differentiation, while NG2-glia help maintain microglial homeostasis and suppress excessive inflammatory responses [131]. For example, NG2-glia secrete transforming growth factor-β2 (TGF-β2), which acts through microglial transforming growth factor beta receptor 2 (TGFBR2) to sustain CX3C chemokine receptor 1 (CX3CR1) expression and limit microglial activation [132]. Loss of NG2-glia disrupts this pathway, resulting in microglial overactivation and exacerbated neurotoxic inflammation in PD [132].

Extracellular α-syn activates microglial TLRs, initiating inflammatory signaling cascades [33]. α-Syn aggregates also bind microglial cluster of differentiation 11b (CD11b) integrin, generating mitochondrial ROS via Rho/Rho-associated coiled-coil containing protein kinase (ROCK) signaling, thereby shaping the neuroinflammatory environment [133]. Oligodendrocyte damage leads to myelin loss, impairing neuronal conduction and survival.

Microglia-derived TNF-α, IL-1β, and IL-6 contribute to oligodendrocyte injury, whereas anti-inflammatory cytokines (IL-4, IL-10, TGF-β) and growth factors released by M2-like microglia activate survival pathways that promote remyelination and repair [133]. Conversely, oligodendrocytes influence microglia by producing IL-6 in PD, a cytokine capable of enhancing microglial inflammatory activation [16].

This reciprocal activation creates a feed-forward cycle that intensifies neuroinflammation and accelerates neurodegeneration in PD. Understanding microglia–oligodendrocyte interactions offers important opportunities for developing therapeutic strategies that modulate glial reactivity and preserve neuronal and myelin integrity.

## 6. New Therapies Targeting Glial Cells for PD Treatment

Parkinson’s disease remains incurable, underscoring the ongoing need for effective therapeutic strategies. Accumulating evidence indicates that chronic neuroinflammation plays a pivotal role in PD progression. Activated microglia and astrocytes release pro-inflammatory cytokines and cytotoxic mediators that exacerbate dopaminergic neuronal loss and accelerate disease advancement [134]. Although oligodendrocytes are primarily responsible for myelinating neurons, they are also susceptible to the inflammatory milieu; thus, protecting these glial cells and modulating their responses has emerged as a promising therapeutic avenue [134,135] (Table 1).

One major line of investigation focuses on inhibitors of inflammatory pathways. The NLRP3 inflammasome, a key immune complex involved in microglial activation, has been extensively studied in PD models. The selective NLRP3 inhibitor MCC950 effectively reduces microglial activation and suppresses inflammasome activity [147]. Another pharmacological strategy repurposes glucagon-like peptide-1 (GLP-1) receptor agonists, known for their anti-inflammatory and neuroprotective properties. NLY01, a brain-penetrant pegylated derivative of the diabetes drug exenatide, was evaluated in a 36-week placebo-controlled trial in early PD to determine whether reduction of microglial activation could slow disease progression [151]. Therapeutic development increasingly targets intracellular signaling cascades underlying microglial activation and astrocyte-induced neurotoxicity, with several candidates in preclinical stages and a subset progressing to clinical trials.

Gene therapies offer additional strategies to modulate glial-driven inflammation in PD. Viral gene delivery of anti-inflammatory cytokines has demonstrated therapeutic potential. For example, adeno-associated virus (AAV)–mediated microglia-specific expression of IL-10 resulted in localized nigral IL-10 release, reduced α-synuclein aggregation, and preserved dopaminergic neurons in an SNCA-overexpressing mouse model [152].

Cell-based therapeutic approaches, traditionally aimed at replacing lost neurons, may also modulate the neuroinflammatory landscape [153]. A hyaluronic acid–based nanoreinforced hydrogel incorporating GDNF and mesenchymal stem cells was developed to target neuroinflammation in PD. This system reduced microglial activation and enhanced anti-inflammatory gene expression, suggesting its potential as an immunomodulatory therapeutic platform [150].

In parallel, classical immunomodulatory strategies have gained traction in PD therapy. Immunotherapy can selectively target molecules involved in inflammatory signaling. For instance, treatment with a functional anti-TLR2 antibody in a high-expressor α-synuclein mouse model significantly reduced neuronal and astroglial α-synuclein deposition, as well as astroglial IL-6 and other pro-inflammatory mediators [154]. However, prasinezumab, a monoclonal antibody directed against aggregated α-synuclein, failed to demonstrate significant effects on clinical or imaging measures of PD progression in the phase 2 PASADENA trial [155].

Collectively, therapeutic strategies that target glial cells through small molecules, gene delivery, cell transplantation, or immunomodulation constitute a rapidly advancing frontier in PD research. By acting on astrocytes, microglia, and oligodendrocytes, these approaches aim not only to ameliorate symptoms but also to slow or potentially halt the underlying disease process.

## 7. Conclusions and Future Perspectives

In Parkinson’s disease, microglia, astrocytes, and oligodendrocytes form a highly interconnected neuroinflammatory network. Rather than acting as isolated effectors, these glial populations engage in dynamic, bidirectional signaling that can either exacerbate or mitigate neuronal injury. Disruptions of this intercellular communication—driven by dysregulated cytokine and chemokine signaling, oxidative stress–related pathways, and ferroptotic mechanisms—are believed to contribute to the chronic neuroinflammation and progressive neurodegeneration characteristic of Parkinson’s disease. Nonetheless, the precise nature and temporal dynamics of glial–glial interactions in this context remain insufficiently understood. A more refined characterization of microglia–astrocyte, astrocyte–oligodendrocyte, and microglia–oligodendrocyte crosstalk may yield a more integrated view of Parkinson’s disease pathoetiology and help define novel glia-centered therapeutic targets.

Neuroinflammation is now recognized as a hallmark of PD pathology, but it is just one aspect of a complex disease process. Research into the disease’s development highlights various mechanisms, with neuroinflammation being among them. However, there is no consensus that neuroinflammation is the primary mechanism driving the disease. While it plays a critical role in PD and represents a promising target for therapy, it is best understood as part of a network of PD rather than the only factor.

Elucidating glial interactions offers promising avenues for therapeutic intervention. Several emerging strategies aim to modulate maladaptive glial responses, including inhibitors of microglial cytokine release and modulators of pathways that suppress pro-inflammatory signaling in microglia and astrocytes. Additional experimental approaches focus on gene therapies targeting glial cells, interventions designed to prevent the induction of neurotoxic astrocytes or restore astrocyte–oligodendrocyte communication, and cell- or exosome-based therapies intended to deliver neuroprotective or immunomodulatory factors.

A major challenge moving forward will be selectively attenuating chronic inflammation while preserving essential innate immune functions. Combinatorial therapeutic strategies may provide opportunities for more effective disease modification and improved quality of life for individuals with Parkinson’s disease. Ultimately, the therapeutic potential of targeting glial dysregulation and correcting aberrant intercellular interactions may lay the foundation for future disease-modifying treatments.

## Figures and Tables

**Table 1:** Therapeutic strategies targeting neuroinflammation mediated by glial cells in Parkinson’s Disease.

Interventions	Targets	Mechanisms	Experimental Models	Reference
Montelukast	Microglial cysteinyl leukotriene receptor 1 (CysLTR1)	Reduced P2X7 receptor (P2X7R)-mediated neuroinflammation, resynchronized microglial activity, and restored motor function	In vivo: A53T α-synuclein transgenic mice	[136]
Pramipexole (PPX)	Dopamine receptor D3 (DRD3), astrocytic inflammasome complex NLRP3, autophagy	PPX activates DRD3-dependent autophagy in astrocytes, which suppresses NLRP3	In vivo: male C57BL/6 mice underwent bilateral striatal LPS injection, In vitro: astrocyte-specific Atg5 knockdown in vivo	[137]
Kaempferol (KAE)	Microglia and astrocytes in substantia nigra	Suppressesion of the p38MAPK/NF-κB signaling pathway, inhibits pyroptosis (downregulates NLRP3, GSDMD-NT, caspase-1, and ASC), reduces release of IL-1β and IL-18 and decreases iNOS/COX-2	In vivo: 6-OHDA-induced PD rat model, In vitro: LPS-induced BV2 inflammatory cell model	[138]
Ceftriaxone (CEF)	SLC7A11, GPX4 microglia/astrocytes (glial activation), dopaminergic neurons	Inhibits ferroptosis by upregulating SLC7A11 and GPX4, Suppresses activation of glial cells by NF-κB pathway, Reducing neuronal and glial-mediated toxicity	In vivo: MPTP-induced Parkinson’s disease model in mice (C57BL/6) LPS-induced neuroinflammation model In vitro: SH-SY5Y cells treated with MPP^+^ to model dopaminergic neuron injury BV2 microglial cells activated by LPS C8-D1A astrocyte cells activated by TNF-α	[139]
Taurocheno-deoxycholic acid (TCDCA)	Microglia, inflammatory mediators (IL-1β, IL-6, TNF-α); Signaling proteins: TGR5 (Takeda G protein–coupled receptor 5), AKT, NF-κB/IκBα, AMPK, mTOR, PINK1, Parkin	Activates autophagy, Suppresses inflammatory signaling, Improves mitochondrial quality	In vivo: MPTP-induced Parkinson’s disease mouse model. In vitro: BV-2 microglial cells stimulated with LPS.	[140]
NLY01 (long-acting GLP-1 receptor agonist)	Microglial GLP-1 receptor (GLP1R). Microglia-mediated conversion of astrocytes to A1 neurotoxic astrocytes, Astrocytic A1 markers	Activates GLP1R on microglia and reduces microglial secretion of cytokines, Prevents microglia-mediated conversion of astrocytes into A1 phenotype, Protects dopaminergic neurons, reduces α-synuclein pathology, and improves behavioral outcomes	In vivo: α-synuclein PFF mouse model, hA53T α-synuclein transgenic mouse model In vitro: primary neuron, microglia and astrocyte cell cultures.	[141]
Echinacoside (ECH)	Microglial α-synuclein/TLR2/NF-κB/NLRP3 inflammasome axis	Reduces expression of α-synuclein (α-syn) in microglia, suppresses TLR2 activation and downstream NF-κB, and inhibits NLRP3 inflammasome activation	In vivo: MPTP-induced subacute PD mouse model In vitro: BV2 microglial cells treated with α-synuclein + MPP^+^	[38]
BX471 (CCR1 antagonist)	CCR1 receptor	Blocks CCR1, reduces NF-κB activation, lowers expression of pro-inflammatory enzymes/cytokines (iNOS, COX-2, TNF-α, IL-1β), decreases T-lymphocyte infiltration, reduces mast cell chymase and tryptase expression, reduces glial activation, and lowers α-synuclein accumulation	In vivo: MPTP-induced nigrostriatal degeneration in mice	[142]
Ginsenoside Rg1	Iron-regulated proteins in oligodendrocytes	Increases ferritin heavy chain (FTH) expression and decreases ferritin light chain (FTL), helping restore iron homeostasis, reduces lipid peroxidation stress in oligodendrocytes, protects mature oligodendrocytes, and supports myelin sheath integrity	In vivo: Chronic Parkinson’s disease mouse model (MPTP + probenecid)	[23]
Knockdown of lncRNA HOXA11-AS	HOXA11-AS	Reduces HOXA11-AS levels and increases miR-124-3p, miR-124-3p suppresses follistatin-like 1 (FSTL1), reduced NF-κB activation, reduced NLRP3 inflammasome activation, lower pro-inflammatory cytokines	In vivo: MPTP-treated mice, with si-HOXA11-AS delivered to knock down HOXA11-AS In vitro: SH-SY5Y neuronal cells treated with MPTP; BV2 microglial cells stimulated with LPS	[49]
Psoralen	NLRP3 inflammasome	Binds to the NACHT and LRR domains of NLRP3, prevents phosphorylation of NLRP3 at Serine 658, thereby inhibiting inflammasome assembly, and reduces activation of glial cells (microglia and astrocytes)	In vitro: Primary microglia and astrocytes In vivo: MPTP/probenecid (MPTP/p) chronic Parkinson’s disease mouse model	[143]
Chloroquine (CQ)	Autophagy pathway, neuroinflammation	Suppresses abnormal neuronal autophagy, reduces pro-inflammatory cytokines IL-1β and TNF-α, lowers oxidative stress (ROS), and preserves dopamine levels	In vivo: BALB/c mice In vitro: PC12 cells	[144]
OLT1177^®^ (dapansutrile)	NLRP3 inflammasome	Inhibits NLRP3 activation, reduces pro-inflammatory markers, decreases α-synuclein levels, and protects dopaminergic neurons	In vivo: MPTP mouse model of PD In vitro: primary neonatal microglia culture	[145]
Camptothecin (CPT)	Microglia	Activates AKT/Nrf2/heme oxygenase-1 (HO-1) and inhibits NF-κB pathways, reduces pro-inflammatory mediators	In vivo: C57BL/6 mice In vitro: BV-2 microglial cells	[146]
MCC950	NLRP3 inflammasome	Inhibits NLRP3, reducing its expression, decreases microglial activation, reduces immune cell responses, modifies α-synuclein aggregation, protects dopaminergic	In vivo: AAV1/2-mediated overexpression of human A53T-mutant α-synuclein (“hαSYN”) in the mouse substantia nigra	[147]
4-Octyl itaconate (OI)	Microglia	Activates the p62/Nrf2/HO-1/NF-κB axis in microglia, suppresses pro-inflammatory mediators,	In vitro: BV2 mouse microglial cell line	[148]
AAV-mediated delivery of human cerebral dopamine neurotrophic factor (hCDNF)	Dopaminergic neurons, glial cells (microglia, astrocytes), endoplasmic reticulum (ER) stress machinery	Overexpression of hCDNF in striatum (STR) and retrograde transport to substantia nigra, reduces glial inflammation, modulates ER stress, protects nigrostriatal pathway	In vivo: male C57BL/6 mice	[149]
Tocilizumab	Interleukin 6 receptor (IL6R) on neurons	Blocks IL-6 signaling by preventing IL-6 from binding IL-6R, inhibits downstream STAT3 activation	In vitro: astrocytes derived from iPSCs of PD patients	[74]
Supramolecular hyaluronic acid (HA) hydrogel (HG) containing nano-encapsulated GDNF and human mesenchymal stem cells (MSCs)	Microglial cells, inflammatory signaling pathway	The hydrogel reduced NO production, downregulated the expression of pro-inflammatory pathways, upregulated the expression of anti-inflammatory pathways	In vitro: murine microglial cell line BV2	[150]

## Data Availability

No new data were created or analyzed in this study. Data sharing is not applicable to this article.

## Abbreviations

The following abbreviations are used in this manuscript:

PD	Parkinson’s disease
SNpc	Substantia nigra pars compacta
α-syn	α-Synuclein
CNS	Central nervous system
iNOS	Inducible nitric oxide synthase
DAMP	Damage-associated molecular pattern
PRRs	Pattern recognition receptors
TLRs	Toll-like receptors
FcγRs	Fcγ receptors
TLR4	Toll-like receptor 4
NF-κB	Nuclear factor-kappa B
SQSTM1	Sequestosome-1 SQSTM1
IFN-γ	Interferon-γ
TLR2	Toll-like receptor 2
MyD88	Myeloid differentiation factor 88
NLRP3	NOD-like receptor protein 3
LPS	Lipopolysaccharide
TNF-α	Tumor necrosis factor-α
IL-1β	Interleukin-1β
IL-6	Interleukin-6
IL-18	Interleukin-18
CCL2	C-C motif chemokine ligand 2
CD36	Cluster of differentiation 36
PKCδ	Protein kinase C delta
ROS	Reactive oxygen species
IL-4	Interleukin-4
IL-10	Interleukin-10
NADPH	Nicotinamide adenine dinucleotide phosphate
NOX2	NADPH oxidase 2
GSH	Glutathione
GPX4	Glutathione peroxidase 4
ncRNAs	Non-coding RNAs
lncRNAs	Long non-coding RNAs
ceRNAs	Competing endogenous RNAs
miRNAs	MicroRNAs
miR-223-3p	MicroRNA-233-3p
lncRNA GAS5	Long non-coding RNA growth arrest specific 5
miR-124	MicroRNA-124
MEKK3	Mitogen-activated protein kinase kinase kinase 3
GABA	γ-aminobutyric acid
BBB	Blood–brain barrier
MHC-II	Major histocompatibility complex class II
VEGFA	Vascular endothelial growth factor A
NO	Nitric oxide
PFFs	Preformed fibrils
PSMB8	Proteasome subunit beta type-8
GBP2	Guanylate-binding protein 2
JAK	Janus kinase
STAT	Signal transducer and activator of transcription
CXCL1	C-X-C motif chemokine ligand 1
CX3CL1	C-X3-C motif chemokine ligand 1
CD44	Cluster of differentiation 44
CB2R	Cannabinoid receptor 2
Foxg1	Fork head box g1
MAP1LC3B	Microtubule-associated protein 1 light chain 3 beta
MPTP	1-methyl-4-phenyl-1,2,3,6-tetrahydropyridine
NOX4	NADPH oxidase 4
H_2_O_2_	Hydrogen peroxide
MPO	Myeloperoxidase
OPN	Osteopontin
4-HNE	4-hydroxynonenal
AMPK	AMP-activated protein kinase
BDNF	Brain-derived neurotrophic factor
TGF-β1	Transforming growth factor- β 1
GFAP	Glial fibrillary acidic protein
OPCs	Oligodendrocyte precursor cells
MBP	Myelin basic protein
iPSC	Induced pluripotent stem cell
MSA	Multiple system atrophy
MHC-I	Major histocompatibility complex class I
IRP1	Iron regulatory protein 1
TfR1	Transferrin receptor 1
FPN1	Ferroportin
PSAP	Prosaposin
GPR37	G-protein–coupled receptor 37
shRNA	Short hairpin RNA
IGFBPL1	Insulin-like growth factor binding protein like 1
C3	Complement C3
C3aR	C3a receptor
NOD2	Nucleotide-binding oligomerization domain containing 2
RIPK2	Threonine-protein kinase 2
MAPK	Mitogen-activated protein kinase
NLRC5	NOD-like receptor CARD domain containing 5
AKT	Protein kinase B
GSK-3β	Glycogen synthase kinase-3β
TNTs	Tunneling nanotubes
EVs	Extracellular vesicles
MDVs	Mitochondrial-derived vesicles
mtDNA	Mitochondrial DNA
cGAS	Cyclic GMP-AMP synthase
STING	Stimulator of interferon genes
PINK1	PTEN-induced kinase 1
AQP4	Aquaporin-4
IL-1α	Interleukin-1α
CCL5	C-C motif chemokine ligand 5
CXCL8	C-X-C motif chemokine ligand 8
LCN2	Lipocalin 2
CXCR1	C-X-C motif chemokine receptor 1
CXCR3	C-X-C motif chemokine receptor 3
CCR3	C-C chemokine receptor type 3
CCR4	C-C chemokine receptor type 4
CCR5	C-C chemokine receptor type 5
CCR6	C-C chemokine receptor type 6
CXCR2	C-X-C motif chemokine receptor 2
CXCR4	C-X-C motif chemokine receptor 4
CXCR5	C-X-C motif chemokine receptor 5
IL-33	Interleukin-33
Nrf2	Nuclear factor erythroid 2-related factor 2
NRG1	Neuregulin-1
FGF	Fibroblast growth factor
FGF1	Fibroblast growth factor 1
FGF9	Fibroblast growth factor 9
FGFR2	Fibroblast growth factor receptor 2
FGFR3	Fibroblast growth factor receptor 3
SLC7A11	Solute carrier family 7 member 11
NG2-glia	Neuron-glia antigen 2
TGF-β2	Transforming growth factor-β2
TGFBR2	Transforming growth factor beta receptor 2
CX3CR1	CX3C chemokine receptor 1
CD11b	Cluster of differentiation 11b
ROCK	Rho-associated coiled-coil containing protein kinase
GLP-1	Glucagon-like peptide-1
AAV	Adeno-associated virus
CysLTR1	Cysteinyl leukotriene receptor 1
P2X7R	P2X7 receptor
PPX	Pramipexole
DRD3	Dopamine receptor D3
Atg5	Autophagy protein 5
KAE	Kaempferol
CEF	Ceftriaxone
TCDCA	Taurochenodeoxycholic acid
ECH	Echinacoside
FTH	Ferritin heavy chain
FTL	Ferritin light chain
FSTL1	Follistatin-like 1
CQ	Chloroquine
CPT	Camptothecin
HO-1	Heme oxygenase-1
OI	4 Octyl itaconate
hCDNF	Human cerebral dopamine neurotrophic factor
ER	Endoplasmic reticulum
STR	Striatum
MSCs	Mesenchymal stem cells
IL6R	Interleukin 6 receptor
HA	Hyaluronic acid
sTNFRs	Soluble TNF receptors
HG	Hydrogel
SCFAs	Short-chain fatty acids
NEAT1	Nuclear enriched abundant transcript 1
hsCRP	Sensitivity C-reactive protein
GPR43	G protein–coupled receptor 43
miR-26b-5p	microR-26b-5p
S100A2	S100 calcium-binding protein A2
mTOR	Mammalian target of rapamycin
